# Severe Axonal Polyneuropathy Revealing Eosinophilic Granulomatosis With Polyangiitis: A Case Report and Review of the Literature

**DOI:** 10.7759/cureus.105812

**Published:** 2026-03-25

**Authors:** Soumia Ait Ami, Manal Toulite, Yasmina Zakaria, Mohamed Chraa, Nissrine Louhab

**Affiliations:** 1 Neurology, Mohammed VI University Hospital of Marrakesh, Cadi Ayyad University, Marrakesh, MAR

**Keywords:** anca-associated vasculitis, axonal polyneuropathy, eosinophilic asthma, eosinophilic granulomatosis with polyangiitis, vasculitic neuropathy

## Abstract

Eosinophilic granulomatosis with polyangiitis (EGPA), formerly known as Churg-Strauss syndrome, is a rare systemic small- and medium-vessel vasculitis characterized by asthma, hypereosinophilia, and multisystem involvement. Peripheral neuropathy is a recognized neurological manifestation of EGPA and may occasionally present as severe axonal polyneuropathy. We report the case of a 64-year-old male with a 10-year history of asthma who presented with progressive lower limb weakness and paresthesia over three months, which led to wheelchair dependence. Electroneuromyography (ENMG) confirmed a severe axonal sensorimotor polyneuropathy. Laboratory findings showed marked hypereosinophilia (9,030/mm³), strongly positive antineutrophil cytoplasmic antibodies (ANCA), and elevated inflammatory markers. Thoraco-abdominopelvic CT revealed bilateral interstitial and alveolar infiltrates. The diagnosis of EGPA was established according to the American College of Rheumatology (ACR) criteria. Treatment with corticosteroids and cyclophosphamide resulted in significant neurological improvement. This report emphasizes the importance of considering EGPA in asthmatic patients presenting with severe axonal polyneuropathy. Early diagnosis and immunosuppressive therapy are essential to improve outcomes.

## Introduction

Eosinophilic granulomatosis with polyangiitis (EGPA) is a rare necrotizing vasculitis affecting small- and medium-sized vessels [1]. It is almost invariably associated with asthma and peripheral eosinophilia. Although the prognosis has significantly improved with corticosteroids and immunosuppressive agents, severe multiorgan involvement remains a major cause of morbidity and mortality. The disease typically evolves through three phases: a prodromal allergic phase, an eosinophilic tissue infiltration phase, and a systemic vasculitic phase [2]. Neurological manifestations of EGPA may involve both the peripheral and central nervous systems and occasionally include neuro-ophthalmological complications [3]. Neurological involvement is one of the most frequent systemic manifestations and occurs in approximately 60 to 70% of patients [4,5].

Peripheral neuropathy most commonly manifests as mononeuritis multiplex; however, symmetrical axonal polyneuropathy may also occur and can occasionally represent the initial presentation of the disease [6,7]. This form is clinically important as it may lead to significant functional impairment and can delay diagnosis when occurring in isolation. Recent studies emphasize that peripheral neuropathy may precede overt systemic vasculitic features, thereby complicating early recognition [8]. Furthermore, the updated 2022 American College of Rheumatology/European Alliance of Associations for Rheumatology (ACR/EULAR) classification criteria highlight the importance of eosinophilia and vasculitic neuropathy in establishing the diagnosis of EGPA [9]. Although peripheral neuropathy is a well-recognized feature of EGPA, its presentation as severe axonal polyneuropathy as an initial manifestation remains uncommon and may pose a significant diagnostic challenge, particularly in the absence of overt systemic features. We report a case of severe axonal polyneuropathy revealing EGPA and aim to highlight this atypical presentation and its diagnostic implications.

## Case presentation

A 64-year-old male with a 10-year history of treated asthma presented with progressive lower limb weakness and distal paresthesia evolving over three months. The motor deficit had gradually worsened, leading to loss of ambulation and eventual wheelchair dependence. This had occurred in the context of general health deterioration. Neurological examination revealed distal, symmetrical motor weakness predominantly affecting the lower limbs. Muscle strength, assessed using the Medical Research Council (MRC) scale, was graded at 3/5 in ankle dorsiflexion and plantar flexion, and 4/5 in proximal lower limb muscles. Upper limb strength was preserved.

Deep tendon reflexes were reduced in the lower limbs and preserved in the upper limbs. Sensory examination showed decreased sensation, more pronounced distally in the lower extremities. These findings were consistent with a peripheral neurogenic pattern predominantly affecting the lower limbs. Electroneuromyography (ENMG) demonstrated a severe axonal sensorimotor polyneuropathy predominantly affecting the lower limbs (Table 1). Motor nerve conduction studies showed markedly reduced amplitudes in the peroneal and tibial nerves, with relatively preserved distal latencies and mildly reduced conduction velocities, consistent with an axonal pattern.

**Table 1:** Nerve conduction study (NCS) results Findings are consistent with a predominantly axonal sensorimotor polyneuropathy affecting the lower limbs. Normal conduction velocity is ≥50 m/s in lower limbs and ≥55 m/s in upper limbs

Nerve	Stimulation site	Latency (ms)	Amplitude (mV/µV)	Conduction velocity (m/s)	Normal amplitude (mV)
Peroneal motor – right	Ankle	5.9	0.8 mV	35	2–6 mV
Peroneal motor – right	Below Knee	14.6	0.5 mV	-	2–6 mV
Peroneal motor – left	Ankle	5.9	0.8 mV	44	2–6 mV
Peroneal motor – left	Below Knee	10.4	0.8 mV	-	2–6 mV
Tibial motor – right	Popliteal segment	5.8	1.01 mV	37	4–20 mV
Tibial motor – left	Popliteal segment	6.2	0.9 mV	34	4–20 mV
Median motor -right	Wrist	2.7	7.4 mV	61	4–15 mV
Median motor -right	Elbow	6.6	6.7 mV	-	4–15 mV
Median motor – left	Wrist	2.9	8.0 mV	63	4–15 mV
Median motor – left	Elbow	6.8	8.0 mV	-	4–15 mV
Ulnar motor – right	Wrist	2.4	10.9 mV	68	6–12 mV
Ulnar motor – right	Below elbow	5.9	10.5 mV	-	6–12 mV
Ulnar motor – right	Above elbow	7.2	10.1 mV	-	6–12 mV
Ulnar motor – left	Wrist	2.6	10.0 mV	66	6–12 mV
Ulnar motor – left	Below elbow	6.4	10.2 mV	-	6–12 mV
Ulnar motor – left	Above elbow	7.7	10.1 mV	-	6–12 mV
Median sensory –right	Wrist	2.4	36.6 µV	61	≥20 µV (sensory)
Median sensory – left	Wrist	2.6	27.0 µV	62	≥20 µV (sensory)
Ulnar sensory – right	Wrist	2.4	18.7 µV	61	≥17 µV (sensory)
Ulnar sensory – left	Wrist	2.4	19.6 µV	58	≥17 µV (sensory)
Sural sensory – right	Ankle	2.6	9.6 µV	58	≥6 µV (sensory)
Sural sensory – left	Ankle	1.9	8.0 µV	63	≥6 µV (sensory)

Sensory nerve conduction studies revealed reduced amplitudes in the sural nerves bilaterally, while sensory responses in the upper limbs were relatively preserved. Overall, these findings supported a length-dependent axonal neuropathy, more pronounced in the lower extremities, in keeping with a vasculitic process in the appropriate clinical context.

**Figure 1:**
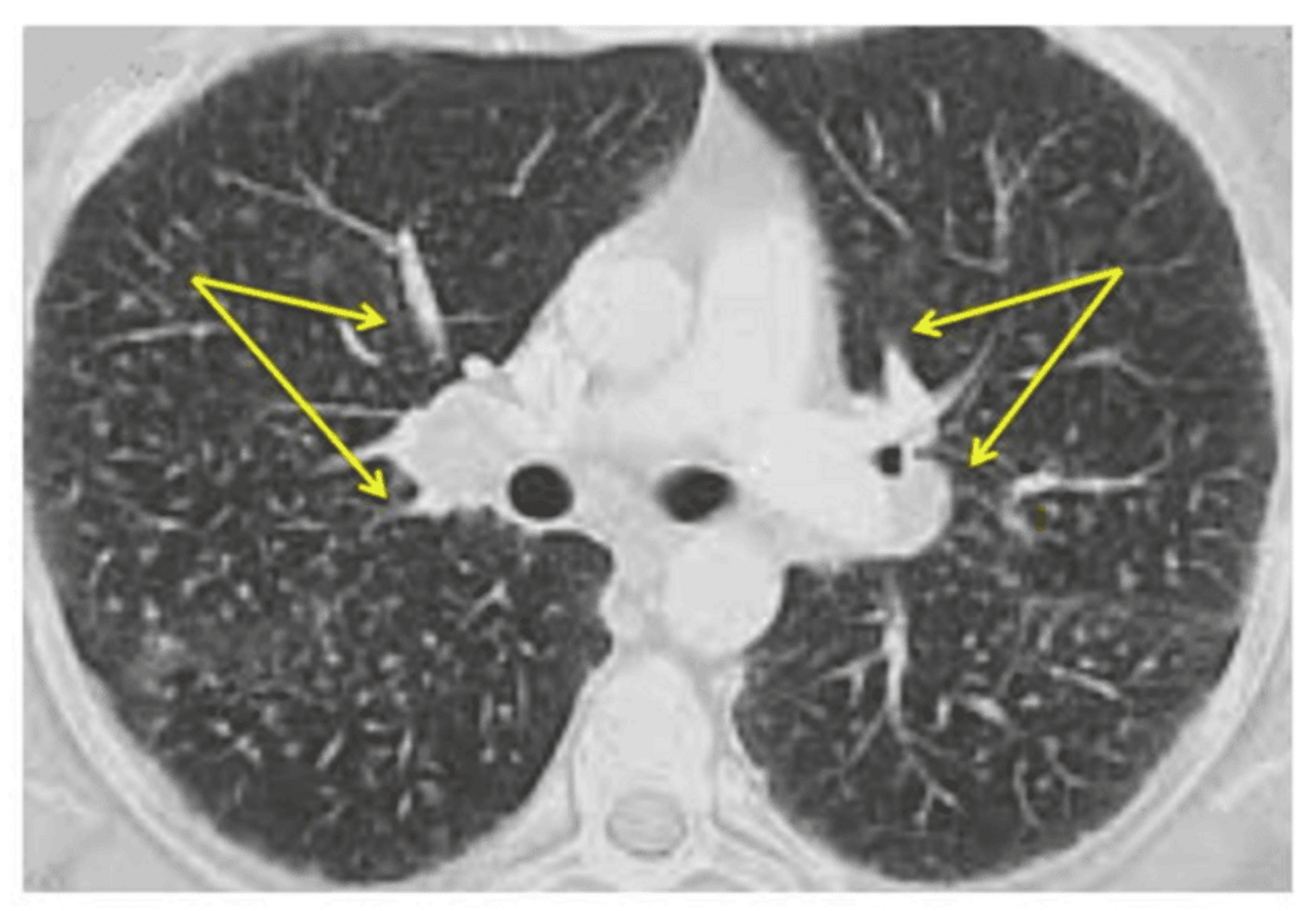
Thoracic CT scan findings The image shows bilateral, patchy interstitial and alveolar infiltrates with predominantly peripheral distribution (yellow arrows), suggestive of eosinophilic lung involvement CT: computed tomography

Laboratory investigations revealed marked hypereosinophilia (9,030/mm³), positive perinuclear anti-neutrophil cytoplasmic antibodies (p-ANCA), and elevated erythrocyte sedimentation rate and inflammatory markers. Vitamin B12 and folate levels were within normal limits, and serum angiotensin-converting enzyme (ACE) was normal. Thoraco-abdominopelvic CT scan showed bilateral, patchy interstitial and alveolar infiltrates with a predominantly peripheral distribution (Figure 1). These findings were suggestive of eosinophilic pulmonary involvement in the appropriate clinical context. The ENT examination was unremarkable.

After excluding metabolic, toxic, infectious, and nutritional causes of neuropathy, the diagnosis of EGPA was established based on the 2022 ACR/EULAR classification criteria. High-dose corticosteroids were initiated, followed by intravenous cyclophosphamide pulses. Progressive neurological improvement, along with normalization of eosinophil count, was observed over subsequent months.

## Discussion

EGPA is a necrotizing vasculitis affecting small- and medium-sized vessels. Its annual incidence ranges from 0.5 to 6.8 cases per million inhabitants [1,2]. Neurological involvement is frequent and represents one of the main systemic complications. Previous studies have described the clinical spectrum and long-term outcomes of patients with EGPA, highlighting the frequency of systemic involvement [4]. This report highlights the importance of considering EGPA in patients presenting with unexplained axonal polyneuropathy, particularly in the presence of asthma and eosinophilia. Peripheral neuropathy occurs in up to 62% of patients and most commonly presents as mononeuritis multiplex due to ischemic damage of the vasa nervorum [5]. However, symmetrical axonal polyneuropathy has also been described and may mimic other inflammatory or metabolic neuropathies [6,7]. Early clinical series have also reported heterogeneous neurological presentations in patients with EGPA [7]. The pathophysiology involves necrotizing vasculitis of the epineurial vessels, leading to ischemic axonal degeneration [5].

Recent case reports have described atypical immunological profiles in EGPA-associated neuropathy, including the presence of anti-myelin oligodendrocyte glycoprotein (MOG) antibodies, suggesting complex neuroimmune mechanisms [8]. Additionally, contemporary literature emphasizes that peripheral neuropathy may represent the first manifestation of EGPA, particularly in asthmatic patients, reinforcing the need for early recognition [8]. According to the updated ACR/EULAR classification criteria, eosinophilia and vasculitic neuropathy are central diagnostic elements for EGPA [9]. Treatment depends on disease severity. Corticosteroids remain the first-line therapy, whereas cyclophosphamide is recommended in severe cases or in the presence of poor prognostic factors [2,10]. Early immunosuppressive therapy significantly improves neurological recovery and reduces relapse risk.

A brief literature review was conducted, including previously reported cases of EGPA-associated neuropathy identified through PubMed using relevant keywords. Only cases with detailed clinical and electrophysiological data were included. A recent case reported by Kawasaki et al. described severe EGPA-related peripheral neuropathy occurring after the discontinuation of mepolizumab, highlighting the potential severity of neurological involvement and the importance of prompt immunosuppressive therapy [10]. A summary of previously reported cases of EGPA presenting with severe polyneuropathy is provided in Table 2. Our case illustrates severe axonal polyneuropathy as the presenting manifestation of EGPA and highlights the importance of early multidisciplinary management.

**Table 2:** Comparison with recently reported cases ANCA: antineutrophil cytoplasmic antibodies; IVIG: intravenous immunoglobulin

Study	Year	Initial presentation	Neuropathy type	ANCA status	Treatment	Outcome
Sehgal et al. [6]	1995	Systemic vasculitis	Mostly mononeuritis multiplex	Variable	Steroids ± cyclophosphamide	Stabilization
Yousuf et al. [8]	2023	Peripheral neuropathy	Axonal neuropathy	Positive	Steroids + immunotherapy	Improvement
Kawasaki et al. [10]	2025	Acute severe neuropathy after the discontinuation of mepolizumab	Severe axonal polyneuropathy with tetraplegia	Negative	Steroids + IVIG + immunosuppressants	Partial improvement
Present case	2025	Severe axonal polyneuropathy	Symmetrical sensorimotor axonal neuropathy	Strongly positive	Steroids + cyclophosphamide	Marked improvement

Strengths and limitations

Strengths

This report highlights an uncommon presentation of EGPA as severe symmetrical axonal polyneuropathy leading to significant functional impairment. The detailed clinical, electrophysiological, and biological assessment strengthens the diagnostic argument. Furthermore, the integration of recent literature enhances the report's relevance and contemporary value.

Limitations

The main limitation was the absence of nerve biopsy confirmation, although the diagnosis was established based on accepted clinical and biological criteria. Additionally, long-term follow-up data remain limited, which prevents the evaluation of relapse risk and sustained neurological recovery.

## Conclusions

EGPA is a rare systemic vasculitis that may present with severe peripheral neuropathy, occasionally as an initial and atypical manifestation. Early recognition of such neurological presentations is essential to avoid diagnostic delay and to initiate appropriate immunosuppressive therapy. This report highlights the importance of considering EGPA in patients presenting with rapidly progressive axonal polyneuropathy associated with eosinophilia and systemic features, even in the absence of overt vasculitic manifestations.
